# Fractionated Radiosurgery Alone for Thirty-seven Brain Metastases: Not Everything that can be Counted Counts

**DOI:** 10.7759/cureus.1985

**Published:** 2017-12-25

**Authors:** Christian Hyde, Shannon Kinser, Christopher Croft, Patricia Schantz, Kayla Brown, Rajendra Vazirani, Jikun Wei, Ioana Bonta

**Affiliations:** 1 Radiation Oncology, Cancer Treatment Centers of America, Newnan, Ga; 2 Radiology, Cancer Treatment Centers of America, Newnan, Ga; 3 Medical Oncology, Cancer Treatment Centers of America, Newnan, Ga

**Keywords:** hypofractionated, single isocenter, lung cancer, stereotactic radiosurgery, frameless radiosurgery, radiation therapy, multiple brain metastases, brain metastases, image guided, fractionation

## Abstract

There is an ongoing debate as to the maximum number of brain metastases that can safely and practically be treated with a single course of radiosurgery. Despite evidence of durable local control and favorable overall survival when treating 10 or more brain metastases with radiosurgery alone, some institutions and guidelines still limit radiosurgery to an arbitrary number of metastases.

As demonstrated by this case report, the number of lesions is not so important when the patient’s life expectancy is otherwise good and body tumors are controllable. In the current era of effective targeted therapies, multi-year survival with brain metastases is increasingly common. Treating 37 brain metastases simultaneously in a five-fraction stereotactic course is technically feasible and in this case, resulted in 100% local and distant control in the brain for 18 months ongoing without any additional brain radiation. We discuss patient selection factors when treating large numbers of brain metastases, and present a possible class solution when using five daily fractions of 6 Gray (Gy) with a single plan and isocenter.

## Introduction

Whole brain radiation therapy is the historical standard for treating multiple brain metastases, but this practice results in worse neurocognitive functions as compared to treatment with radiosurgery alone [1]. A meta-analysis of three randomized trials has also shown worse overall survival in patients under age 50 who were randomized to receive supplemental whole brain irradiation after radiosurgery for one to four brain metastases [2].

Large treatment series of Stereotactic Radiosurgery (SRS) alone by Yamamoto, et al. have shown equivalent local control, neurological outcomes, and overall survival for matched patients with two to nine versus 10 or more brain metastases [3]. Predictors of longer survivals among patients with 10 or more metastases include female gender, younger age, controlled primary, no extracerebral metastases, better Karnofsky Performance Status (KPS) score, better modified recursive partitioning analysis (RPA) class, smaller tumor volume, and higher peripheral dose. Many of these favorable prognostic factors apply to the patient featured in this case report of radiosurgery alone for 37 brain metastases treated in a single course.

## Case presentation

A 44-year-old non-smoking female presented with a three-month history of worsening cough, hemoptysis, shortness of breath, and bilateral hip pain. The chest computed tomography (CT) scan revealed a 4.2 cm right hilar mass with bronchial compression and post-obstructive pneumonia. Bronchoscopic biopsy of the right lower lobe mass was performed, consistent with lung adenocarcinoma, thyroid transcription factor-1 (TTF-1) positive. A positron emission tomography (PET) scan showed extensive spinal and pelvic bone metastases, bulky right lung mass, and bilateral mediastinal lymphadenopathy. The brain magnetic resonance imaging (MRI) revealed 37 enhancing metastases ranging from 3 mm to 16 mm in size. The stage was T3 N3 M1b with extensive bone and brain metastases. Her KPS was 80, with limitations in walking due to pain and having to sleep inclined in a chair due to shortness of breath when supine.

Her young age, female gender, non-smoking status, previously normal health, and desire to continue working as a small business owner were considered in formulating her treatment plan, along with a strong clinical suspicion that she would be a candidate for targeted drug therapy of an epidermal growth factor receptor (EGFR), anaplastic lymphoma kinase (ALK) or c-ros oncogene-1 (ROS) mutation. A recommendation was made for a treatment using the stereotactic technique to minimize impairment of normal brain function. She also consented to radiation to the bulky disease in her chest and pelvis, which served the dual purpose of both palliation and consolidation.

Planning technique

The patient was simulated in a frameless full-face mesh mask, with a CT slice thickness of 1.25 mm. This was fused in the Varian Eclipse planning system (Varian Medical Systems, Inc., Palo Alto, California) with her contrast-enhanced volumetric MRI scan. A three-dimensional brain volume (BRAVO) sequence was performed with a 1.5 Tesla MRI, using a field of view of 26 cm, repetition time of 9.3 msec, echo time of 4.1 msec, and 1 mm between slices.

**Figure 1:**
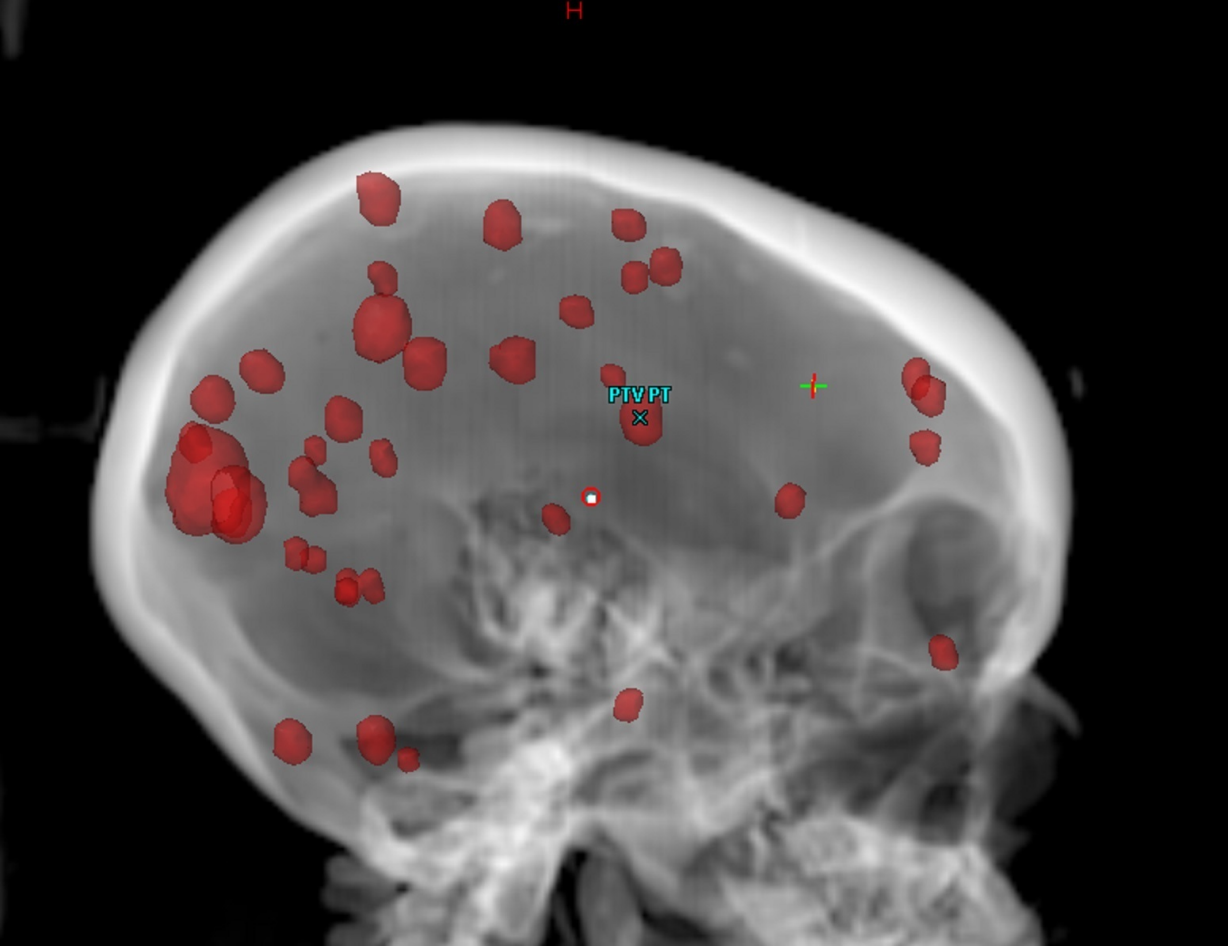
Single planning tumor volume (PTV) with 37 brain metastases

All 37 brain metastases were identified and contoured as a single gross tumor volume (GTV) measuring 4.96 mL in total. A margin of 1 mm was added to create the planning tumor volume (PTV) measuring 11.13 mL in total (Figure 1). 

A single-isocenter volume modulated arc therapy (VMAT) plan was created adopting techniques described by Clark, et al. [4]. Based on arc angles similar to those demonstrated by the Brainlab Multiple Brain Metastases planning module (Brainlab AG, Munich, Germany), four non-coplanar partial arcs were created at 40-degree couch angle intervals: 30, 70, 290, and 330 degrees.

Inverse planning optimization

**Figure 2:**
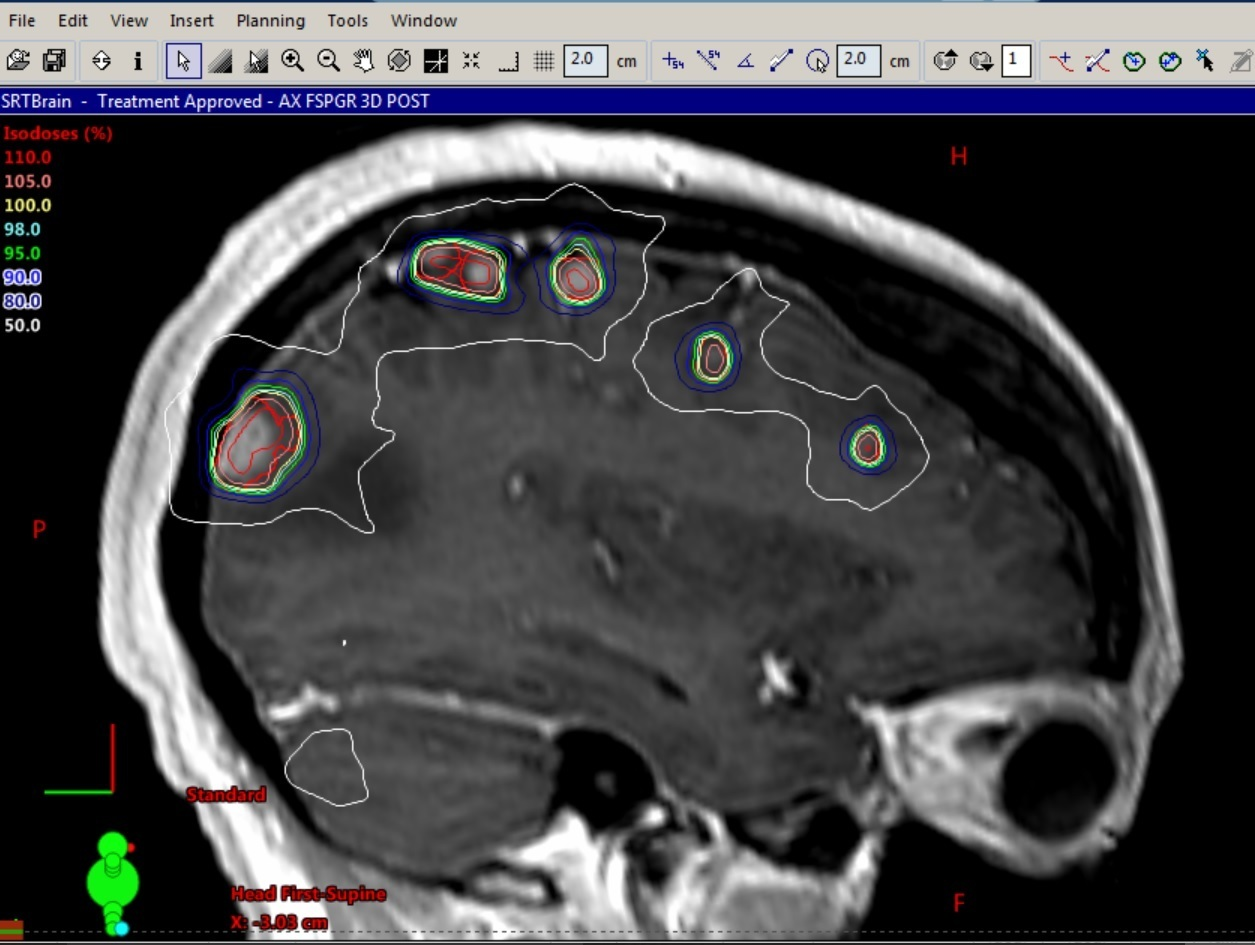
Sagittal isodose plan view showing six metastases

Three concentric shells or rings around the PTV were created: 1-5 mm ring, 6-10 mm ring, and 11-25 mm ring. An outer brain structure (brain minus rings) was generated that includes all normal brain tissue 26 mm or more beyond the PTV. These structures were used to create steep dose gradients in all directions during optimization, ideally 10% per millimeter fall-off from the target. Where tumors closely abut, high dose may bridge across the normal brain, necessitating hand-drawn avoidance structures to displace this dose. A single VMAT plan was generated to treat all 37 brain metastases simultaneously (Figure 2).

Quality assurance for 37 metastases

The clinical policy for performing patient-specific quality assurance (QA) on multiple fraction brain radiosurgery patients involves comparing the dose from an Eclipse verification plan with the dose measured using Sun Nuclear ArcCheck (Sun Nuclear, Melbourne, Florida). Our clinical tolerance for patient-specific QA is a gamma passing rate ≥ 95% with 3%/2mm and a 10% threshold. For this patient, during the irradiation of the ArcCheck, 890 diodes were used for measurement, of which 880 passed, resulting in a 98.9% passing rate at 3%/2mm with a 10% threshold. Beyond the standard patient-specific QA, routine machine QA is performed including a daily Winston-Lutz test with a 0.5 mm mean deviation tolerance and 1 mm maximum deviation tolerance; a weekly picket fence test was performed to evaluate the multi-leaf collimator (MLC) positioning accuracy; and since the rotation error is even more critical for single isocenter, multi-lesion treatments, a test of the isocentricity of our imaging isocenter was performed using Varian IsoCal with a tolerance of 0.2 degrees and 0.3 mm.

Five-fraction radiosurgery

The dose was prescribed such that 95% of the PTV received 30 Gray (Gy) in five daily fractions of 6 Gy each (5x6 Gy), with a maximal dose of 119.9%. Our usual maximum dose constraint for such cases is 125% or 37.5 Gy. The patient was treated with a Varian TrueBeam linear accelerator using 10 megavoltage (MV) photons, which produce less dose to the scalp than 6 MV photons. A daily cone-beam CT scan was obtained for image guidance, and sub-millimeter alignment was achieved using a six degree of freedom robotic couch. The median time of treatment delivery was 13 minutes and 7 seconds from the start of image-guided cone-beam CT to the end of the fourth arc, including the time needed to make four couch angle changes.

Extracranial treatment

**Figure 3:**
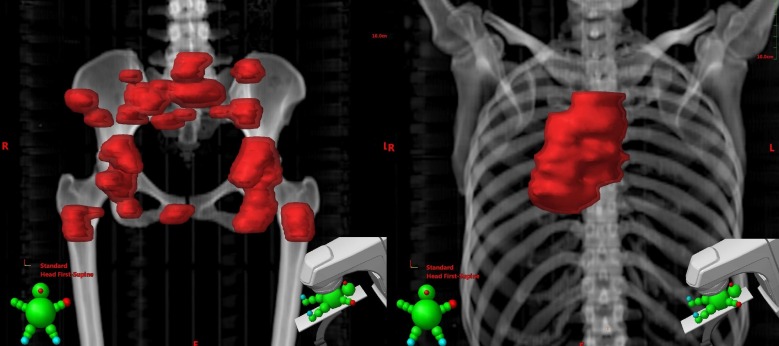
Pelvis planning tumor volume (PTV) with 23 positron emission tomography (PET) avid metastases and right lung, nodes, and T4-T7 spine

Following radiosurgery completion, radiation therapy was given to the patient’s bulky and symptomatic body tumor sites. For the pelvis, a deformable registration was made in a MIM Maestro workstation (MIM Software Inc., Cleveland, Ohio) using the patient’s simulation CT and diagnostic PET scan, then all pelvic metastases with a standardized uptake value of four or greater (SUV4+) were auto-contoured using a customized algorithm. After manual refinements to the SUV4+ contour, a 5 mm margin was added to generate the pelvic PTV, which received eight daily fractions of 3.5 Gy each for 28 Gy total. The treatment was delivered to her 23 pelvic bone metastases with a Tomo3D plan (Accuray Inc., Sunnyvale, California). The Tomotherapy unit delivers radiation via a helical technique, yielding an extremely conformal dose pattern without necessitating inverse-planned intensity modulated radiation therapy (IMRT) and its associated expense. Her bulky right lung primary, mediastinal nodes and T4-T7 spine received five doses of 4 Gy each, also using a Tomo3D plan (Figure 3). 

Tumor molecular analysis

Molecular tumor analysis confirmed EGFR exon 19 deletion, which is more frequently seen in young, non-smoking females, and is associated with increased overall survival. She was started on afatinib, a second-generation tyrosine kinase inhibitor targeting this EGFR mutation.

## Discussion

Central nervous system control

**Figure 4:**
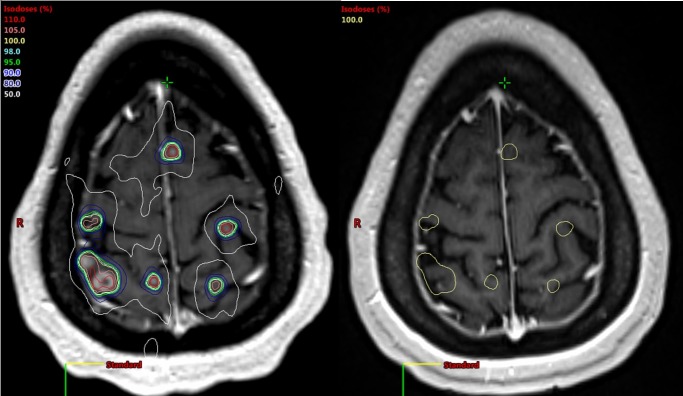
Baseline brain magnetic resonance imaging (MRI) with plan isodose lines, and an 18-month MRI with control of all treated lesions

Compared to her pre-treatment MRI below, the patient continues to have 100% local control of all treated brain metastases, which appear smaller or resolved on her 18-month MRI (Figure 4). There are no new brain metastases on her 18-month MRI, without any need for re-treatment to the brain. This favorable outcome with a single course of radiosurgery questions the traditional belief that whole brain irradiation is needed to treat micro-metastases in the brain, and suggests that even in the presence of 37 gross brain metastases from lung cancer, brain control can be maintained in some patients by radiosurgery alone.

Systemic control

**Figure 5:**
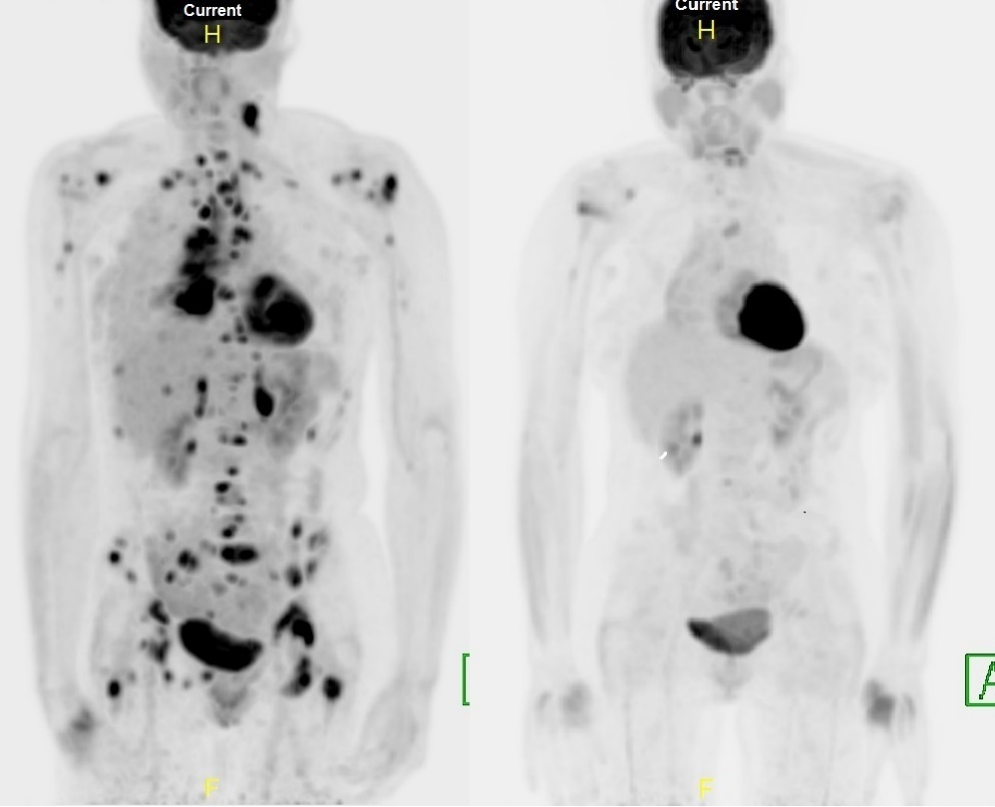
Baseline and four-month positron emission tomography-computed tomography (PET-CT) scans

Palliative radiation therapy was given to the lung and pelvic disease, with relief of the patient’s obstructive pneumonia and hip pain, while simultaneously consolidating these major reservoirs of systemic disease. As seen below, the patient’s initial PET scan shows later complete metabolic response in all irradiated areas on her four-month follow-up PET (Figure 5). Her L4 spine and bilateral shoulders were un-irradiated and still showed some mildly persistent activity.

She developed bone-only progression in un-irradiated sites at 15 months, and new genomic analysis of the patient’s plasma showed the EFGR exon 19 deletion was still present, but in addition, she had developed an echinoderm microtubule-associated protein like 4 to ALK (EML4-ALK) gene rearrangement. At the recommendation of her molecular tumor board, crizotinib treatment was added concurrently to her afatinib therapy.

Of great importance to the patient, she has accomplished her goal of continuing to run her demanding business these past 20 months, without cognitive impairments.

Impact of fractionation

When treating large volumes of the normal brain, as with bulky, numerous or adjacent tumors, a short course of five fractions benefits from the four R’s of radiobiology: repair of normal tissue, reoxygenation of tumor hypoxia, redistribution of the tumor into radiosensitive cell cycle stages, and yet is too short for tumor repopulation. As an example of repair, the optic chiasm tolerance increases from roughly 8 Gy in one fraction to 25 Gy in five fractions. A fifth R of immune recruitment may also be at work, as Dewan, et al. have shown the immune-mediated abscopal effect of radiation with three doses of 8 Gy or 5x6 Gy, but not with a single fraction of 20 Gy [5].

A five-fraction schedule for brain metastases has been evaluated by many groups, including two German centers that treated up to four brain metastases and found that five fractions of 7 Gy (5x7 Gy) gave a 46% complete response rate and overall local control of 87%. For patients with prior or anticipated whole-brain irradiation, the radiosurgery dose was reduced to 5x6 Gy. Toxicity was more common in the group receiving 5x6 Gy or 5x7 Gy than in those receiving 10 fractions of 4 Gy (22% vs. 0% toxicity), showing the protective effect of even further fractionation [6].

Higher dose schedules have been investigated by others but with an increased risk of necrosis. Lischalk, et al. evaluated 5x6 Gy, 5x7 Gy, and five fractions of 8 Gy (5x8 Gy) for single brain metastases >2 cm diameter or located in eloquent areas. Both patients treated with 5x8 Gy developed radionecrosis. Five fractions of 7 Gy resulted in 100% local control but symptomatic necrosis in two of 13 patients (15%), both of whom required subsequent surgery. No necrosis was found among five patients treated with 5x6 Gy, but two of these patients failed locally, one of whom had a pontine metastasis, which location may have constrained the dose. GTV size did not correlate with the risk of radionecrosis, but maximal dose did (p = 0.042) [7].

Re-planning our patient’s case using a single fraction SRS dose of 20 Gy produced a normal brain volume at 12 Gy (V12) of 45 mL, which is 5.7 times higher than the V12 of 7.9 mL found by Blonigen, et al. to predict for symptomatic radionecrosis after linear accelerator-based single SRS. For such cases, they recommend fractionated radiosurgery [8].

Number versus volume

The ability to achieve 100% local control in 37 metastases without any symptomatic edema, necrosis, or steroid dependence suggests that the number of brain metastases is less important than the volume of tumor requiring a high effective dose.

Even using 5x6 Gy, a low dose in some series, our plan did not meet some published constraints, as the patient’s volume of the normal brain (brain minus GTV) receiving 28.8 Gy was 11.2 mL. This is over the 7 mL reported by Inoue, et al. as a threshold for radiation necrosis when treating large single tumors up to 4 cm diameter in five fractions for 31 to 35 Gy total [9]. Our favorable result may suggest that dose constraints for necrosis based on large solitary metastases treated to 35 Gy might not translate exactly to the situation of dozens of small metastases treated to 30 Gy. Keeping the maximum dose relatively low in our patient's case may also have had a protective effect against necrosis, as suggested by Lischalk, et al. [7]. Local control was probably helped by most lesions being sub-centimeter in diameter, each lesion thus requiring a lower dose to control than a bulky tumor.

Importance of systemic control

It has been known since the first Radiation Therapy Oncology Group (RTOG) RPA, two decades ago, that brain metastasis patients with controlled systemic disease generally outlive those with a normal uncontrolled systemic disease. A controlled primary was found to be a major predictor of survival, second only to having a KPS of 70 or higher [10]. Because systemic control is not a static variable, but can be improved by judicious use of radiation therapy, consolidative body radiation should be considered when the patient’s long-term prognosis warrants optimism.

## Conclusions

Five-fraction radiosurgery is a safe, effective, and well-tolerated method of treatment for multiple brain metastases. Fractionation may allow treatment of more metastases than might be possible with single fraction SRS, due to reduced risk of radiation necrosis. Durable 100% local and distant control in the brain is possible after radiosurgery alone, even in a patient presenting with 37 brain metastases, when there is an effective systemic therapy option. Control of tumors in the body is a major predictor of control in the brain, and the systemic origin of brain metastases should not be overlooked as the root cause of delayed brain relapse. 

The focus on the number of brain metastases as a selection criterion for radiosurgery is a historical artifact of what was once a labor-intensive process per metastasis but ignores the weightier matters of tumor biology, advances in targeted and immune agents, patient function, and quality of life. In our current era, the number of brain metastases does not matter.
